# Clean synthesis of biolubricant range esters using novel liquid lipase enzyme in solvent free medium

**DOI:** 10.1186/s40064-015-0937-3

**Published:** 2015-04-07

**Authors:** Jayati Trivedi, Mounika Aila, Chandra Dutt Sharma, Piyush Gupta, Savita Kaul

**Affiliations:** Biofuels Division, CSIR-Indian Institute of Petroleum, Mohkampur, Dehradun, UK 248005 India; Analytical Sciences Division, CSIR-Indian Institute of Petroleum, Mohkampur, Dehradun, UK 248005 India

**Keywords:** Biolubricant, Lipase, Hydrolysis, Enzymatic esterification, Biocatalysis

## Abstract

In view of the rising global problems of environment pollution and degradation, the present process provides a ‘green solution’ to the synthesis of higher esters of lubricant range, more specifically in the range C_12_-C_36_, using different combinations of acids and alcohols, in a single step reaction. The esters produced are biodegradable in nature and have a plethora of uses, such as in additives, as lubricating oils and other hydraulic fluids. The enzymatic esterification was performed using liquid (non-immobilized or free) lipase enzyme, without any additional organic solvent. Soluble lipase proves to be superior to immobilized enzymes as it is more cost effective and provides a faster process for the production of higher esters of lubricant range. An interesting finding was, that the lipase enzyme showed higher conversion rates with increasing carbon number of straight chain alcohols and acids. Reactions were carried out for the optimization of initial water concentration, temperature, pH of the substrate mixture and the chain length of the substrates. Under optimized conditions, the method was suitable to achieve ~ 99% conversion. Thus, the process provides an environment friendly, enzymatic alternative to the chemical route which is currently used in the industrial synthesis of lubricant components.

## Background

Lubricants have an enormous market worldwide and its consumption has increased by leaps and bounds in the last decade. On the other hand, according to IEA World energy outlook, at current rate of procurement and depletion, the fossil fuels may succumb to the demands and dry out in 53 years from now. These two contradictory factors have brought about renewed interest in the use of bio-based materials as a source of lubricants. Emphasis on the development of renewable, biodegradable, and environment friendly industrial lubricants has resulted in the widespread use of natural oils and fats for non-edible purposes.

Lubricants have a myriad of applications and a huge market potential. The main problems encountered in the conventional lubricants made from mineral oil are their non-fossil origin, toxicity and poor biodegradability. According to an estimate, 50% of the lubricant that is sold around the globe ends up in the ecosystem due to spillage, incidents, evaporation or similar reasons, which can cause toxicological effects if accumulated in the environment (Willing 2001; Horner 2002).

The term biolubricants applies to lubricants from plant-origin, which are both, rapidly biodegradable and non-toxic to humans and other living organisms, especially in aquatic environments. Biodegradability provides an indication of the persistence of the substance in the environment and is the yardstick for assessing the eco-friendliness of substances (Salimon et al. 2010). Biodegradable esters have been of interest, for example, as surgical implants and agricultural plastic films (Linko et al. 1995). Europe and USA are the global leaders in the production of environment-friendly lubricant solutions.

Lipases (triacylglycerol acylhydrolase, EC 3.1.1.3) are enzymes which belong to class hydrolases. Their natural function is to catalyze hydrolysis of triacylglycerols (fats) in vivo and form glycerol and fatty acids. Fats have poor solubility in aqueous systems and create an interface with water. Therefore, these enzymes have been evolved to work optimally in an organic-aqueous interface through a phenomenon called interfacial activation (Sarda and Desnuelle 1958; Anthonsen et al. 1995; Verger 1997). Another advantage of using lipases from microbial sources, including bacteria, fungi and yeast, is that they do not need any co-enzymes for their activity, and thus have a simple mechanism of action which can be easily understood and controlled.

Lipases have the potential to catalyze various kinds of reactions such as synthesis of esters, triacylglycerols and polymers, enantiomer resolution, fatty-acid enrichment, biodiesel production, phospholipid conversion, carbohydrate modification etc. Due to their multiple uses, lipases have recently garnered interest of researchers across the globe and is one of the most studied groups of enzymes.

Their use in ester production has been studied by various researchers in the past. Production of trimethylolpropane esters of rapeseed oil fatty acids by immobilized lipase Rhizomucor miehei Lipozyme IM 20 has been discussed by Linko et al. (1997). Production of biolubricants for low temperature applications from trimethylolpropane (TMP) and carboxylic acids from C_5_ to C_18_ using different heterogeneous catalysts like silica-sulfuric acid, Amberlyst-15, and immobilized lipase B from *Candida Antarctica* has been studied and compared with Akerman et al. (2011a).

The use of immobilized lipases for long chain ester production has been extensively reported in the past. Most of the existing literatures mention the use of immobilized enzyme Novozym 345, IM20 for the esterification purpose, which results in rise in the cost of the process (Akerman et al. 2011b). In some other reports immobilization of the powdered enzyme was done, which is itself a tedious task and involves high expenses (Ghamgui et al. 2004). This makes the overall process economically non-competitive against the chemical processes using catalyst.

Literature available on the use of soluble lipases is scarce. In one of the study, biodiesel production using the liquid lipase enzyme NS81006 in oil/water biphasic system was carried out and mechanism study of methanolysis of Triglyceride was done (Lv et al. 2010). A patent has revealed the process of biodiesel production from fatty acid feedstock using liquid lipase enzyme (Austic et al. 2014). In yet another report, phospholipases have been combined with a liquid lipase for one-step biodiesel production using crude oils. This allows performing the enzymatic degumming and transesterification in a single step, using crude soybean oil as feedstock, and converting part of the phospholipids into biodiesel (Cesarini et al. 2014).

There are some reports available which elucidate the use of Novozymes Callera Trans L enzyme for the purpose of FAME (Fatty acid methyl esters) production. This enzyme has been used for the production of FAMEs in water containing systems using crude soyabean oil as a feedstock (Cesarini et al. 2013). In a recent report, kinetic model for the enzymatic transesterification of rapeseed oil with methanol using Callera TM Trans L was discussed using Ping Pong Bi-Bi mechanism (Price et al. 2014).

Considering the limited literature available demonstrating the use of liquid lipase enzyme Callera Trans L in the production of the higher esters of lubricant range, and their potential to address the cost effectiveness and rate of the reaction issues that plaqued previous studies; the current work aims to test the suitability of liquid lipase in catalyzing ester production and further optimizing the reaction parameters for an economical and efficient process.

## Materials and methods

### Materials used

The liquid lipase enzyme used in the reactions was the new soluble lipase Callera Trans L (*Thermomyces lanuginosus*, EP 258068). All the acids and alcohols used for esterification reactions were purchased from Sigma Aldrich and were chromatographically pure. Other chemicals used in the study were obtained from standard sources. As the reference samples of esters in the carbon range C_12_- C_36_ were not available in the market, pure esters were synthesized using acidic chemical catalyst.

### Experimental procedures

An integrated assembly was constructed for carrying out the esterification reactions at different temperature conditions. The reaction was carried out in a 250 ml round bottom flask dipped in a water bath whose temperature was varied as desired in the reaction parameters. The weight of the reactants was chosen in the range of 30–50 gms, depending on their molecular weights, for maintaining the mole ratio as 1:1 (acid:alcohol) in all the experiments. A constant stirring at 200 rpm was provided to the reaction mixture for better mixing. A vacuum pump was attached to the reaction flask for the simultaneous removal of water produced during the reaction. Continuous removal of water produced during the esterification reaction was done for driving the reaction in the forward direction.

The initial pH of the different reactant combinations of acids and alcohols was in the range of 2–3. For adjusting the pH of the reactants, sodium hydroxide pellets were ground to obtain the powdered form. They were then added to the reactant mixture and kept for stirring till all the sodium hydroxide got dissolved. Subsequently, the enzyme was added to the reaction mixture.

### Product purification and analysis methods

Aliquots of volume 1 ml were withdrawn at intervals of 6 hrs from the start of the reaction. There was no by-product formation, so the sample purification could be done in a single step by removing sodium hydroxide and enzyme from these samples. The aliquots were water washed (0.5 ml) 3 times for the removal of NaOH (added for adjusting pH) and enzyme. The purified samples were used for titrimetric calculations. Samples were then passed through a basic alumina column having 1 cm diameter. The amount of adsorbent alumina was taken according to the acid value of the sample calculated in 2.3.1. The eluted samples from the column were acid free and were used for TLC, HPLC and NMR analysis.

#### Titrimetric calculations

The amount of residual acid in the reaction mixture is a measure of the extent of the reaction. The residual acid (mg KOH/g) in the reaction mixture was calculated using titrimetric data. Acid value titrations were performed as described in the ASTM-D-974/11 using a Metrohm743, titrando, auto titrator equipped with a model 801 stirrer. The titration endpoint was determined by the instrument and visually verified using a phenolphthalein indicator. Each sample was run in duplicate and mean values were reported.

#### Thin layer chromatography (TLC) analysis

The initial detection of the ester formed during the reaction was done using thin layer chromatography technique. Silica gel 60 F_254_ and 5% ethyl acetate in n-hexane were selected as the stationary phase and the mobile phase respectively. The purified sample (mentioned in 2.3) and the reference samples (mentioned in 2.2) were diluted in 1:5 ratio with acetone and the spots of these samples were placed adjacent to each other on the base line of a TLC plate. After running the samples for 20 mins on the plate, detection of the final position of the spots was accomplished by drying the TLC plate for 10 mins and subsequently placing it in an Iodine vapor container for 5 mins.

#### High pressure liquid chromatography (HPLC) analysis

The quantitative analysis of long chain esters in the purified samples (mentioned in 2.3) was done by chromatographic analyses using an Agilent 1260 series analytical system equipped with an evaporating light scattering detector (ELSD) 1260 infinity. The HPLC system was run at 40 psi pressure and 50°C drift temperature using Agilent eclipse plus C18 column. 96% methanol and 4% hexane were used as the mobile phase.

**Figure 1:**
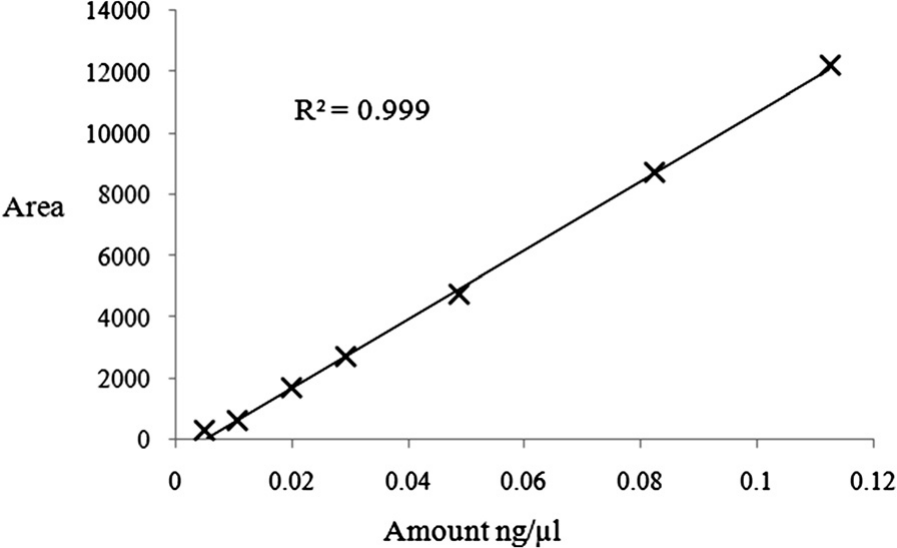
**HPLC Calibration curve obtained from the pure oleyl oleate synthesized via catalytic route.**

Reference samples of esters synthesized via. chemical catalyst route (mentioned in 2.1) were used for generating a calibration curve. The pure reference sample of ester of known concentration was diluted to prepare samples which varied in the concentration range 0.005 to 0.12 ng/ul. These diluted samples of different known concentrations were injected into HPLC to obtain the corresponding area. Thus, a calibration curve with high correlation index (linear data fitting) was obtained for each ester. This calibration curve was used to calculate the ester yield (%) in the purified product obtained from esterification reaction. One of the calibration curves for the reference ester - oleyl oleate synthesized via catalytic route is shown in Figure 1.

#### NMR analysis

The ^13^C NMR spectra of the sample were recorded on a Bruker Avance III NMR spectrometer equipped with a 5 mm-mm BBFO probe resonating at the frequency of 125.7 MHz using 30% (w/v) in CDCl_3_ solutions. Quantitative ^13^C spectra were acquired using the NOE suppressed, inverse gated proton decoupled technique (Waltz-16), with a sweep width of 19 kHz. 2 k number of scans were collected using a 5 sec relaxation delay. All the ^13^C spectra were processed with 1.0 Hz line broadening prior to FT.

The purified samples as mentioned in 2.3 were used for the analysis. All the ^13^C NMR spectra were referenced to tetramethylsilane (TMS) at 0 ppm. Before starting the analysis, the spectra obtained were corrected for phase and baseline and then each of them was separated into different regions that correspond to different types of protons and carbons according to their position in the molecule.

## Results and discussion

### Thin-layer chromatography (TLC) results

**Figure 2:**
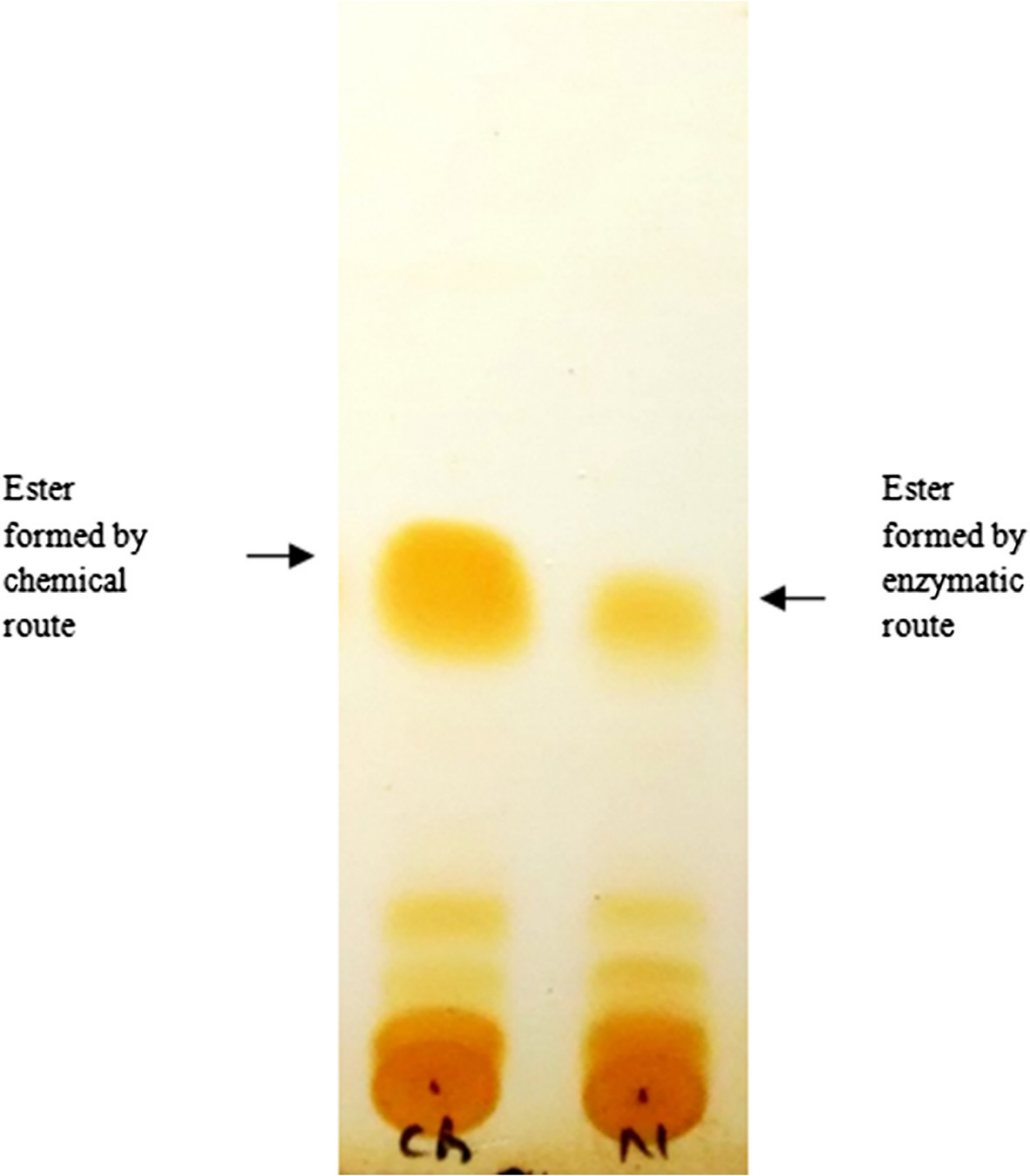
**TLC images for initial detection of ester production using enzymatic route.** 5% ethyl acetate was used as the mobile phase and the samples were run to observe their final position on the plate.

As the mobile phase traveled up the TLC plate, the compounds present in the samples also moved up at different rates according to the nature of the compounds. A preliminary confirmation of the presence of ester in the product was established by observing the same final position of the reference compound and the reaction product after traveling up the TLC plate (Figure 2).

### High pressure liquid chromatography results

**Figure 3:**
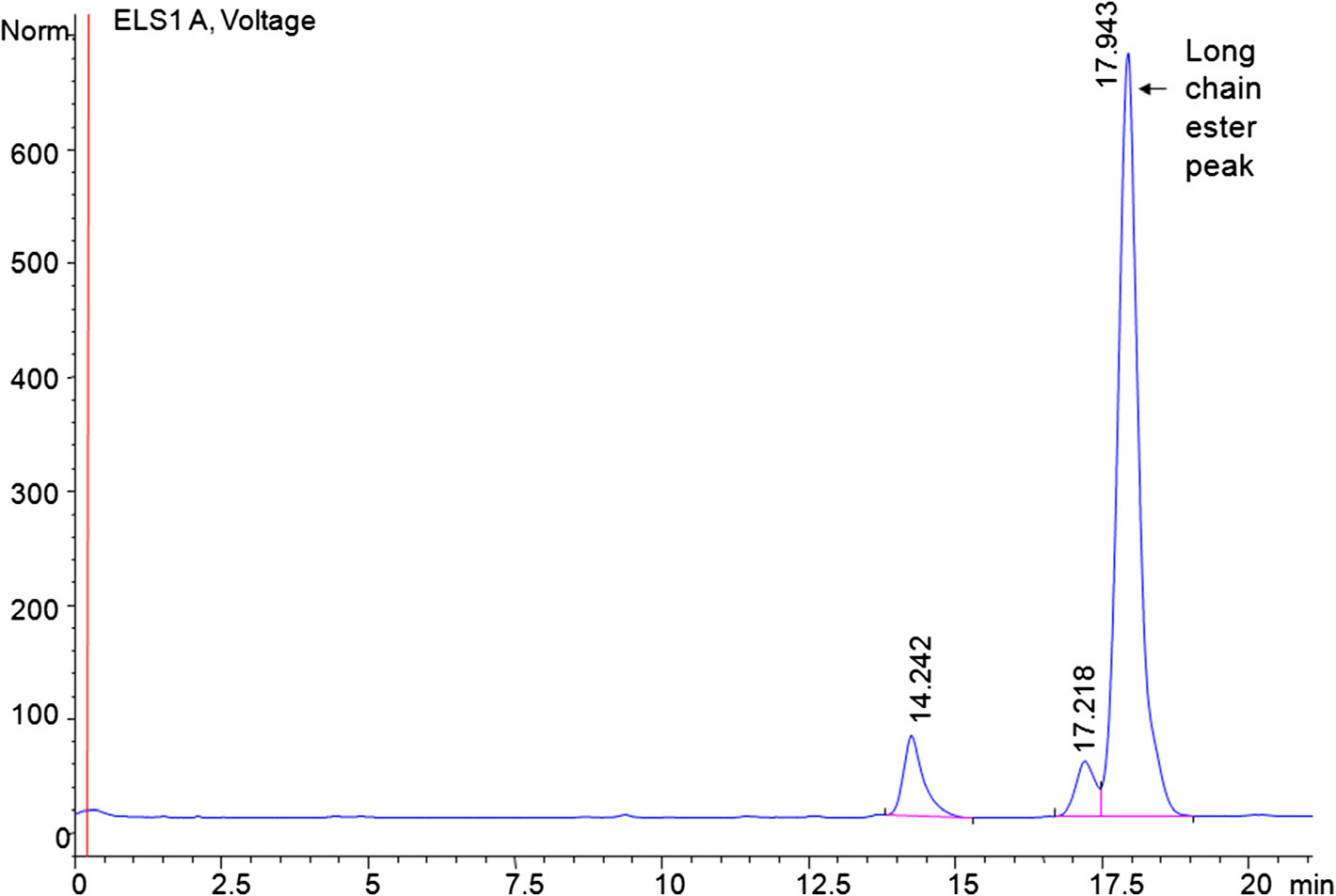
**HPLC chromatogram of the purified product of esterification reaction.** The major peak shows the long chain ester oleyl oleate synthesized via enzymatic route.

The HPLC chromatogram of the enzymatic reaction products matched exactly with the reference esters, confirming the formation of ester in the product. In HPLC chromatogram (Figure 3), the major peak corresponds to the long chain ester oleyl oleate. Similarly, HPLC chromatograms were obtained for all the esters synthesized using different reactant combinations (not shown). The percentage yield of esters formed in all the experiments was calculated using the calibration curve. The percentage esterification calculated by both HPLC analysis (which showed ester formation) and titration calculations (which showed residual acid) were found to be complementary to each other.

### NMR results

**Figure 4:**
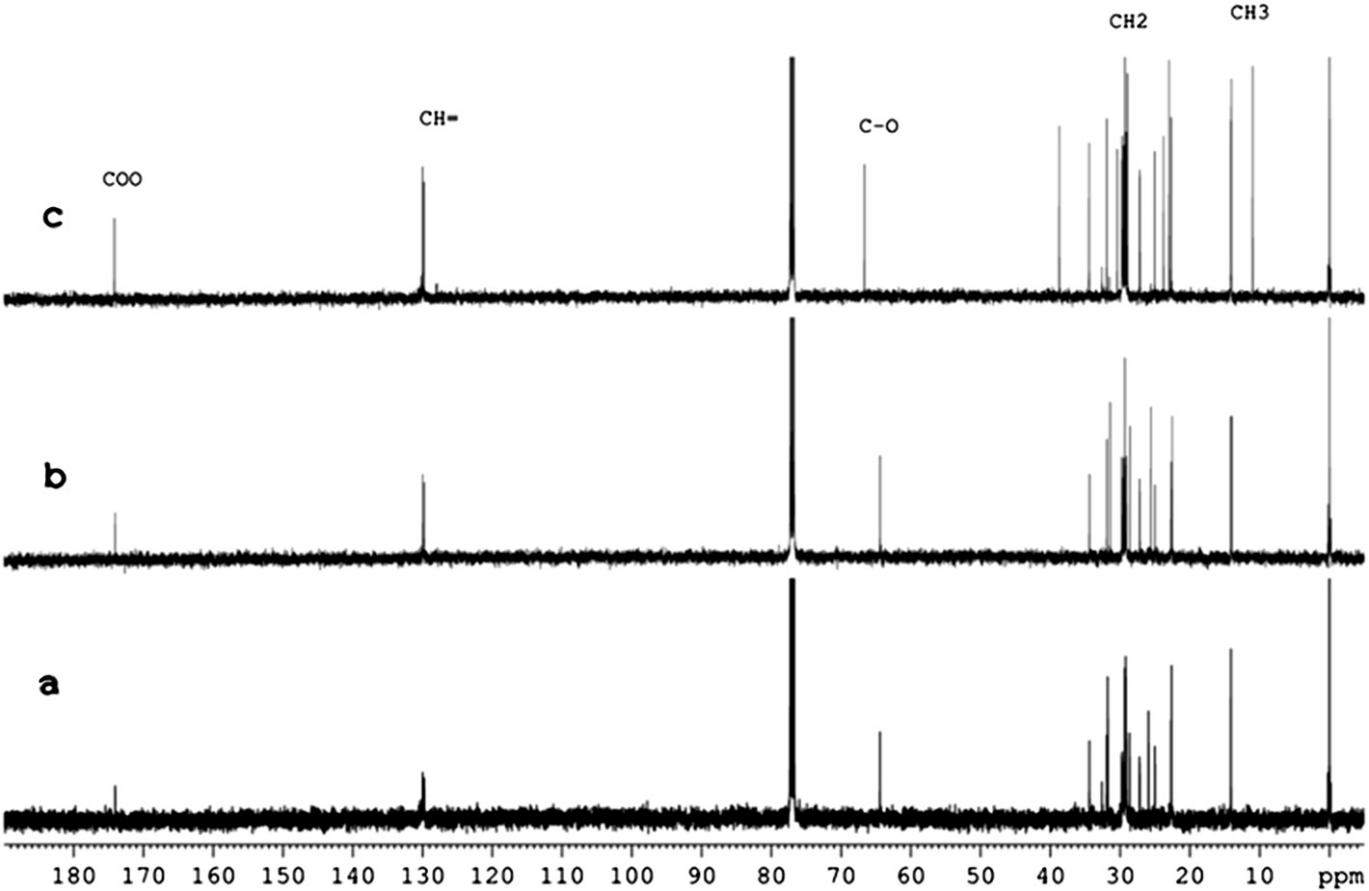
**NMR spectra of the products obtained after esterification reaction.** Spectra confirms the presence of ester in the product obtained when oleic acid is treated with **a)** Octanol **b)** Hexanol **c)** 2-Ethyl hexanol.

NMR spectra of the products obtained from the esterification reaction further confirmed the nature of the compound. The data from Figure 4 shows the ^13^C NMR spectra of 3 different esters obtained when oleic acid is treated with Octanol (marked as ‘a’), Hexanol (marked as ‘b’), 2-Ethyl hexanol (marked as ‘c’). The peak obtained between 170–180 ppm corresponding to the -COO- group was detected. Other peaks corresponding to the carbon types –CH=, −CO-, −CH_2_-, −CH_3_ were also detected. Due to the presence of branched chain in 2-ethyl hexanol, two peaks corresponding to the carbon of -CH_3_ group can be seen in the spectra marked as (c). Overall spectra confirms the formation of long chain esters.

### Effect of water

Several reports in the past have reported the importance of control of water content in the lipase catalyzed reactions. Water greatly affects the enzyme action by interacting with the non-covalent bonding and hydrogen bonds present inside the enzyme. Water molecules play a number of critical roles in enzyme catalysis, including mass transfer of substrates and products, nucleophilicity and proton transfer at the active site, and solvent shell-mediated dynamics for accessing catalytically competent conformations (Brogan et al. 2014). Low water content usually reduces enzyme activity. High water content can also decrease reaction rates by aggregating enzyme particles and causing diffusional limitations. Although a minimum quantity of water is necessary to enhance the lipase activity, best yield of ester is obtained when the water is present in restricted quantity (Yahya et al. 1998). Ester synthesis and hydrolysis are reversible processes, and according to the Le Chatelier’s principle, the reaction equilibrium can be shifted in the desired direction of ester production, either by taking excess of one of the substrates or by restricting the water content of the reaction system.

**Figure 5:**
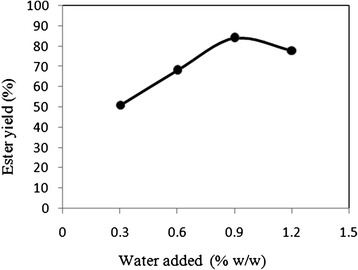
**Effect of external water added at the start of the reaction on the ester yield.** The other reaction conditions were: Temperature 30°C, acid:alcohol mole ratio 1:1, enzyme added 1% w/w and pH 7.

The lipase enzyme used in this study was examined at various levels of hydration. The water produced during the reaction was simultaneously removed using the vacuum assembly. At the same time, water was added externally to the reaction mixture before the start of the reaction to enhance the initial activity of the enzyme. The effect of initial water on enzymatic activity was examined by adding water ranging from 0.3% to 1.2% (w/w) to the reaction mixture. The plot in Figure 5 shows the initial water added to the reactant system at time t = 0 hr and its effect on the ester yield. The other reaction conditions were; Temperature 30°C, acid:alcohol mole ratio 1:1, enzyme added 1% w/w and pH 7. The reaction was carried out between oleic acid and oleyl alcohol and was stopped after 24 hrs. Ester yield was calculated using HPLC data. It was observed that on increasing the water content, the yield also increased and a maximum yield was observed at 0.9% w/w of water content. On increasing the water content above that, the ester yield gradually decreased. This can be explained by the fact that increasing the water content above a certain level shifts the reaction equilibrium in the reverse direction and favors hydrolysis reaction.

### Effect of pH

pH of the reactants is a key parameter in optimizing the product yield. Variations in the pH of the medium, results in changes in the ionic form of the active site and can cause changes in the activity of the product and hence the yield of the product formed. Changes in pH may also alter the three-dimensional shape of the enzyme (Shuler and Kargi 1997). For these reasons, enzymes are only active over a certain pH range. Thus, experiments were performed to find out the pH of the reactant mixture which corresponds to maximum ester yield.

**Figure 6:**
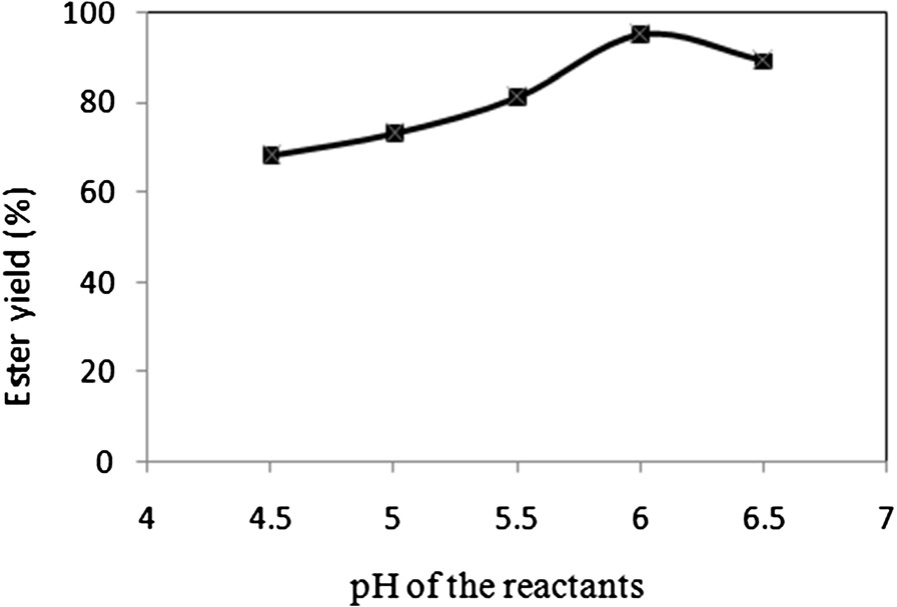
**Effect of pH of the reactants on the ester yield.** The other reaction conditions were: Temperature 30°C, acid:alcohol mole ratio 1:1, enzyme added 1% and external water added 0.9% w/w.

For finding out the optimum pH for the lipase activity, initial pH of the reactant solution was varied in the range 4.5 - 6.5 by the method described in 2.2. The temperature of all the reactions in determining the optimum pH was 30°C and the enzyme added was 1% of the total reactant weight (including the weight of Sodium hydoxide and water) and external water added 0.9% w/w. The reaction was stopped after 18 hrs, as the ester yield does not increase considerably after that. The ester yield was calculated using HPLC. Data from Figure 6 clearly shows that the enzyme showed its best activity at a pH of 6. Decreasing or increasing the pH from 6 resulted in decreased enzyme activity. Based on the results of these measurements, pH of reactants was adjusted to 6, using the same method as above, in further experiments.

### Effect of temperature

The effect of temperature was studied in a series of experiment in the range 20 - 60°C. The other reaction conditions were: acid:alcohol mole ratio 1:1, enzyme added 1% w/w, external water added 0.9% w/w and pH 6. In lipase catalyzed reactions, temperature significantly influences both the initial rate of the reaction and stability of the enzyme. The reactants used for this set of reaction were hexanol and hexanoic acid. The other reaction conditions like initial water content, molar ratio and enzyme concentration were kept same for all sets of reactions. The reaction was stopped at 18 hrs as the yield of product does not increase considerably after that time. The samples were purified as mentioned in 2.3 and ester yield was calculated using HPLC data.

**Figure 7:**
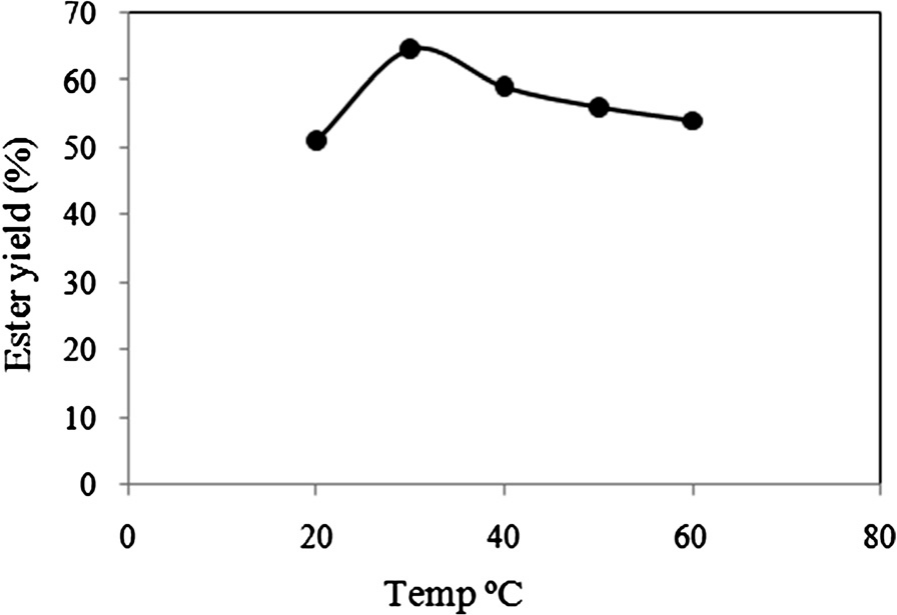
**Effect of variation of the reaction temperature on the ester yield.** The other reaction conditions were: acid:alcohol mole ratio 1:1, enzyme added 1% w/w, external water added 0.9% w/w and pH 6.

The enzyme was found to be thermostable and did not deactivate upto 60°C. However, it can be observed from Figure 7, that the best enzyme activity was obtained at 30°C. The enzyme activity decreased on further increasing the temperature. This property of the enzyme is advantageous for us as it enables us to carry out the reaction at lower temperature and thus makes the process more energy efficient. Based on the finding, 30°C temperature was used in further experiments.

### Effect of type of substrate

In determining the effect of chain length and chain type of the alcohols on the yields of ester formed, reactions were carried out with different combinations of alcohols and acids. In all the reactions, the conditions were as follows: 1:1 acid/alcohol mole ratio, 1% enzyme, 30°C temperature, 0.9% w/w external water added and pH 6. The reaction was stopped at 18 hrs as the product yield does not increase considerably after that, in any of the reactions. Ester yield was calculated using HPLC data. For clear comparison, oleic acid was kept constant in all the reactions and the alcohols were varied. The combination of reactants used in reaction sets A to E are given in Table 1. The effect of different alcohols in reaction sets A to E, on the yield of ester is described in 3.7.1 and 3.7.2.

**Table 1:** **Reactant combinations used in reaction sets A to E and the yield of ester obtained**

**Reaction set**	**Reactants used**	**Structure of ester formed**	**Ester yield (%)**
A	Oleyl alcohol and Oleic acid		99
B	Octanol and Oleic acid		86
C	Hexanol and Oleic acid		82
D	3, 7 Di methyl 1-Octanol and Oleic acid		64
E	2- Ethyl hexanol and Oleic acid		53

### Effect of chain length of alcohol

**Figure 8:**
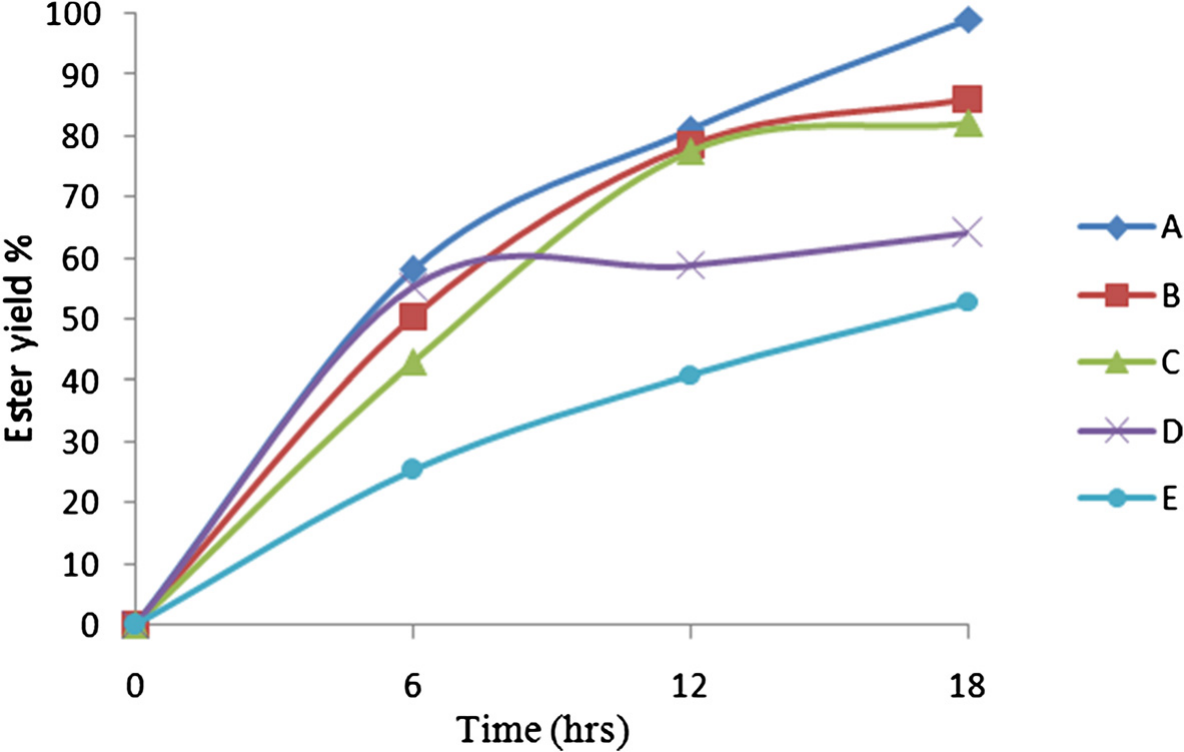
**Effect of substrate chain on the ester yield.** Figure shows the ester yield (%) at different time points, corresponding to six different reactant sets A to E mentioned in Table 1. The other reaction conditions were: Temperature 30°C, acid:alcohol mole ratio 1:1, enzyme added 1% w/w, external water added 0.9% w/w and pH 6.

Maximum yield of 99% was obtained in set A when Oleyl alcohol was used as the alcohol. Comparing the results (Figure 8) obtained for reaction sets A, B and C it is clearly visible that, when the chain length of alcohol decreased the ester yield also decreased. Thus, it can be concluded that the activity of enzyme for ester production was higher when higher carbon chain reactants were used.

### Effect of branching in alcohol

The yield of ester formed was less in the case of reaction sets D and E as compared to the reaction sets A, B and C. It can also be deduced from Figure 8 that when branched alcohols were used, the ester yield decreased drastically. Another observation was that, when an ethyl group was present in the branching (in reaction set E) it further reduced the ester yield as compared to the methyl group branching (in reaction set D). This observation can be attributed to the higher steric hindrance created by the ethyl group as compared to the methyl group which lowered the activity of the enzyme.

## Conclusion

The described process can be used for successful production of esters, without the presence of any solvent, making the process environment friendly. The biggest advantage of the present process is that the esterification reactions can be carried out at the room temperature with significant yield of the desired product. Moreover, the study shows that, there is no by-product formation, which makes the purification of ester simpler. Also, this liquid enzyme is unique in the sense that the ester yield gets increased with the increasing chain length of the reactants, which makes the production of long chain biolubricants more economical. Thus, in view of all the advantages stated above, the soluble lipase used in this study is clearly more advantageous than the immobilized lipase enzyme or chemical catalysts, which are commonly used for this purpose. Under optimized reaction conditions elucidated in the present work, the present process can provide a faster, economical and eco-friendly alternative for the large scale industrial production of long chain biodegradable esters for lubrication purpose.
